# Correlation of DNA load, genotyping, and clinical phenotype of *Mycoplasma pneumoniae* infection in children

**DOI:** 10.3389/fped.2024.1369431

**Published:** 2024-04-09

**Authors:** Wei Wang, Lu Wang, Zhaoqing Yin, Shujuan Zeng, Guohua Yao, Yuqiao Liu, Yulian Fang, Cuian Ma, Hualei Cui

**Affiliations:** ^1^ Tianjin Pediatric Research Institute, Tianjin Children’s Hospital (Tianjin University Children’s Hospital), Tianjin, China; ^2^ Tianjin Key Laboratory of Birth Defects for Prevention and Treatment, Tianjin, China; ^3^ Division of Neonatology, The People’s Hospital of Dehong Autonomous Prefecture, Mangshi, Yunnan, China; ^4^ Division of Neonatology, Longgang District Central Hospital of Shenzhen, Shenzhen, Guangdong, China; ^5^ Infectious Diseases Section, Tianjin Children’s Hospital (Tianjin University Children’s Hospital), Tianjin, China

**Keywords:** *mycoplasma pneumoniae*, children, DNA load, genotyping, clinical characteristics

## Abstract

**Introduction:**

This study aimed to investigate the correlation between *Mycoplasma pneumoniae* (MP)*-*DNA load in the bronchoalveolar lavage fluid (BALF) of children with MP pneumonia (MPP) and its subtypes, relevant laboratory data, imaging, extrapulmonary complications in infected children, and its clinical significance in evaluating the disease.

**Methods:**

Children hospitalized with MPP at Tianjin Children's Hospital between December 2017 and December 2020 were selected for the study, excluding those with mixed viral, bacterial, and fungal infections. Children were divided into low- and high-load groups according to the MP DNA load in BALF using real-time quantitative fluorescence polymerase chain reaction (PCR). After a successful MP culture, positive specimens were subjected to PCR-Restriction fragment length polymorphism and Multiple-locus variable number tandem repeat analysis typing. Basic data, clinical information, laboratory data, and radiological results were collected from all children included in the study.

**Results:**

The PI-I type dominated the different load groups. Children in the low-load group had more wheezing and shortness of breath; however, children in the high-load group had a higher length of hospitalization, maximum fever temperature, higher chills/chilliness, incidence of abdominal pain, and higher C-reactive protein (CRP), procalcitonin (PCT) and aspartate aminotransferase (AST) levels. Children in the high-load group were more likely to have imaging changes such as pleural effusion, and the incidence of respiratory infections and extrapulmonary complications was higher than that of those in the low-load group. We applied Spearman's correlation analysis to clarify the relationship between MP DNA load and the clinical severity of MPP. We found that MP DNA load was positively correlated with length of hospitalization, maximum fever temperature, CRP, PCT, Interleukin-6 (IL-6), and AST levels, and negatively correlated with fever and cough durations, white blood cell count (WBC), and proportion of monocytes (MONO). The degree of correlation was as follows: length of hospitalization > IL-6 > cough duration > AST > fever duration > PCT > WBC > proportion of MONO > maximum fever temperature > CRP levels.

**Conclusions:**

MP DNA load was not correlated with MP typing but was significantly correlated with the children's clinical phenotype. Therefore, the MP DNA load helps in the early diagnosis of infection and can better predict disease regression.

## 1 Introduction

*Mycoplasma Pneumoniae* (MP) is an important pathogenic microorganism causing community-acquired pneumonia in children. Owing to its independent survival between bacteria and viruses, its drug resistance rate has risen sharply in recent years, with an annual increase in incidence (1). Previous clinical experience often suggests that MP pneumonia (MPP) can be self-limiting. However, due to the incomplete development of children's tissues, organs, and immune system functions, MP infection can progress quickly and easily, leading to severe pneumonia, extrapulmonary complications (2), and multiple organ failure (3). Furthermore, multiple factors, such as local signs of wheezing and dyspnea combined with systemic infectious poisoning symptoms, cause children to have poor prognoses and high disability and mortality rates. The corresponding sequelae can affect children's daily academic performance, life, and quality of life even if they survive (4, 5). Therefore, early screening and precise assessment of the harm caused by MP infection provide crucial objective clinical predictive guidance for the treatment, prognosis, and well-being of infected children.

In previous studies, serological methods were mainly used to diagnose MP infection, and the detection of immunoglobulin M (IgM) antibodies in the acute phase or the increased immunoglobulin G antibody titers in the acute and recovery phases were the main indicators of current infection. However, for infants and young or immunocompromised children, the sensitivity of serological methods is low because IgM antibodies are associated with their immune status, and antibodies are not often detected (6). Real-time fluorescence quantitative polymerase chain reaction (PCR) has been used to detect the load of MP DNA in the bronchoalveolar lavage fluid (BALF) of children in the control, non-MPP and MPP groups compared with the positivity rate of MP antibody in a single serum. The false-positive and false-negative rates detected using BALF as a sample were significantly lower than those detected using serum, suggesting that using BALF as a test specimen provides higher accuracy. The same group of data also suggests that MP DNA in BALF has a positive rate of 88% in the early stage of the disease (1–7 days), which is significantly higher than the 9% positive rate of serum MP antibody within the same period, indicating that using BALF as a specimen provides a higher degree of accuracy in the early diagnosis of MPP (7). Notably, some researchers have also demonstrated that the detection rate of MP infection can be significantly improved by using the BALF MP DNA test, and the BALF MP DNA results are not affected by the duration of the disease (8).

Quantitative fluorescence detection of MP DNA can quantify the level of MP DNA in the body and accurately reflect the infection and replication of MP in a child's body. Clinicians can also infer the severity of a child's condition based on the copy number of MP DNA and control the medication dosage to achieve the desired therapeutic effect (9). Furthermore, local MP DNA load can objectively reflect the balance between the pathogen and host immune clearance capacity (10). There are currently few studies on the correlation between local MP DNA load in the respiratory tract and clinical severity in children with MP globally. Similar studies have been conducted in China. However, the specimen types are mostly sputum and nasopharyngeal swabs (11–14). Studies have shown a higher false-negative rate of MPP due to the predominantly dry cough, low secretion, and sputum in the early stage of the disease, and poor sputum compliance in pediatric patients, leading to the mixing of saliva in the specimen (15). The upper respiratory tract of the healthy population also carries MP, leading to a higher false-positive rate of the specimen compared with the specimen from the BALF (16). Therefore, BALF can be obtained directly from the diseased tracheobronchial mucosa compared with serology, sputum, and nasopharyngeal swabs as test samples to confirm the diagnosis of MPP. This can minimize the contamination rate of the specimen and is more objective, direct, and independent of the disease duration. However, there has been no clear conclusion regarding using BALF as a sampling specimen in children (7, 17). Therefore, it is necessary to conduct more in-depth and detailed research on the relationship between MP DNA load and clinical severity in the BALF of children infected with MP to obtain more objective conclusions.

This study aimed to analyze MP DNA load and genotyping data, clinical manifestations, relevant laboratory data, imaging, and extrapulmonary complications of infected children to explore the relationship between MP load and the severity of children's clinical conditions and provide a basis for monitoring and predicting MPP.

## 2 Materials and methods

### 2.1 Study participants

This study included children with MP of any sex, aged 2 months to 15 years old, admitted and hospitalized at Tianjin Children's Hospital between December 2017 and May 2020.

The inclusion criteria were clinical signs and symptoms of pneumonia at the time of admission, including fever, cough, abnormal lung auscultation, pulmonary infiltrates on chest radiography, positive serologic test results (serum anti-MP IgM titer ≥1:160 or antibody titer increase ≥four-fold), and positive MP PCR test of BALF (13).

The diagnosis of refractory MPP (RMPP) is based on the presence of clinical signs such as persistent fever, severe cough, dyspnea, and worsening of symptoms on lung imaging with extrapulmonary complications after ≥7 days of regular treatment with macrolide antimicrobials (18).

The exclusion criteria were (1) Tuberculosis and other respiratory pathogen infections confirmed by sputum or blood culture, real-time fluorescence quantitative PCR, antibody detection, and loop-mediated isothermal amplification (LAMP). (2) Underlying diseases such as asthma, chronic cardiopulmonary, congenital, or immunodeficiency diseases (19, 20).

BALF was collected from all enrolled children at an early admission stage, and the collection standards followed the Chinese Guidelines for diagnosing and treating MP in children (18, 19).

Ethical approval was obtained from the Medical Ethics Committee of the Tianjin Children's Hospital (reference number TJWJ2021QN049). This study complied with the principles of the Declaration of Helsinki.

### 2.2 MP DNA testing

Real-time fluorescence quantitative PCR was used to detect MP DNA in the BALF of children, and Sun Yat-sen University Daan Gene Co., Ltd provided the reagent kit. The ABI 7500 gene detection system was used for PCR amplification of the specimen DNA and data processing. Negative quality control, positive quality control, and positive quantitative reference (1.0 × 10^5^, 1.0 × 10^6^, 1.0 × 10^7^, and 1.0 × 10^8^ gene copies/ml, respectively) were used for each experiment. The number of cycles required for the specimen or standard to reach the critical threshold (Ct value) was obtained based on the fluorescence intensity curve, with Ct ≥ 30.0 negative and Ct < 30.0 positive. MP positivity was determined when the assay result was >1.0 × 10^3^ gene copies/ml. According to the level of DNA load, it was categorized into low- (10^3^/ml < MP DNA ≤ 10^6^/ml) and high-load groups (MP DNA > 10^6^/ml) (20, 21).

### 2.3 MP culture

Notably, 200 μl of BALF specimen was inoculated in 1 ml of MP liquid medium [Mycoplasma medium CM0403 (OXOID, UK); glucose and phenol red (Sigma, USA); penicillin G (Amresco, USA); and fetal bovine serum (ExCell Bio, Shanghai Ekosai Bioproducts Co., Ltd.)], mixed well and then set to incubate at 37°C in a warm box. The positive control was also set. The medium initially indicated MP growth if it changed from red to yellow and the bacterial liquid was transparent and not turbid. The specimens confirmed positive using the 200 μl of liquid medium were aspirated and inoculated in the Mp solid medium [Mycoplasma medium CM0401 and yeast extraction (OXOID, UK); glucose (Sigma, MA, USA); yeast extract (Hebei Mali Food Co., Ltd.); fetal bovine serum (ExCell Bio, Shanghai Ekosai Bioproducts Co., Ltd.); and penicillin G (Amresco, USA)]. The inoculated medium was tilted to evenly spread on the petri dish, which was placed in a 5% carbon dioxide environment culture at 37 °C. The plate was observed once daily with a low-power microscope. After about 15 days, typical “fried egg-like” colonies appeared, indicating the survival of MP culture.

The corresponding liquid positive culture was placed in an ultra-low temperature refrigerator at −80°C for numbering and storage for future use.

### 2.4 MP DNA extraction

After the successful MP culture, nucleic acids were extracted using a Nucleic Acid Extraction or Purification Kit (Zhongshan Daan Gene Co., Ltd.). The operation was performed strictly according to the instructions, and the extracted nucleic acids were stored at −20°C and used for subsequent typing studies. The Smart 32 automatic nucleic acid extractor was used.

### 2.5 P1-restriction fragment length polymorphism genotyping

Specimens tested positive by real-time PCR were further typed using PCR-restriction fragment length polymorphism (RFLP) analysis (22).

### 2.6 Multiple-locus variable number tandem repeat analysis genotyping

Based on the primers published by Dégrange et al. (23), five variable number tandem repeat regions (Mpn1, Mpn13, Mpn14, Mpn15, and Mpn16) in the MP gene were amplified using PCR. The amplification products were sequenced, and the sequencing results were analyzed to classify MP into different combinations of the number of repeats in each locus, according to 26 types (A-Z).

### 2.7 Positive reference strain of MP

The international standard FH (ATCC15531) strain of MP type II used in this study was preserved in our laboratory.

### 2.8 Clinical data collection

Basic data, clinical information, laboratory data and radiological results were collected from all children included in the study.

### 2.9 Statistical analysis

SPSS 22.0 software was used for data processing and statistical analysis, and the measurement data were expressed as $\bar{x}$ ± s, and a *t*-test was used to compare the groups. The count data were expressed as a number of cases or percentages, and the chi-square test was used to compare the positive rates in each group. The measurement data conforming to skewed distribution after the normality test were expressed as M (Q1, Q3), and the Mann-Whitney rank sum was used to compare the groups. Spearman's correlation analysis was used to study the degree of correlation and association between the MP DNA load and the observed indicators. Statistical significance was set at *P *< 0.05.

## 3 Results

### 3.1 Basic information

In total, 915 MP DNA-positive specimens from BALF were obtained after real-time fluorescence quantitative PCR testing, and the co-infection with other pathogens was excluded. Among the positive specimens, 465 (50.8%) were male, with a mean age of 6.0 years (4.0, 8.0), and 450 (49.2%) were female, with a mean age of 6.0 years (5.0, 8.0). The male-to-female ratio was approximately 1:1, with a minimum age of 2 months and a maximum of 15 years. According to the MP DNA load grouping, 211 (23.1%) and 704 (76.9%) children were in the low- and high-load groups, respectively.

### 3.2 Relationship between DNA load and genotyping of MP

**Figure 1:**
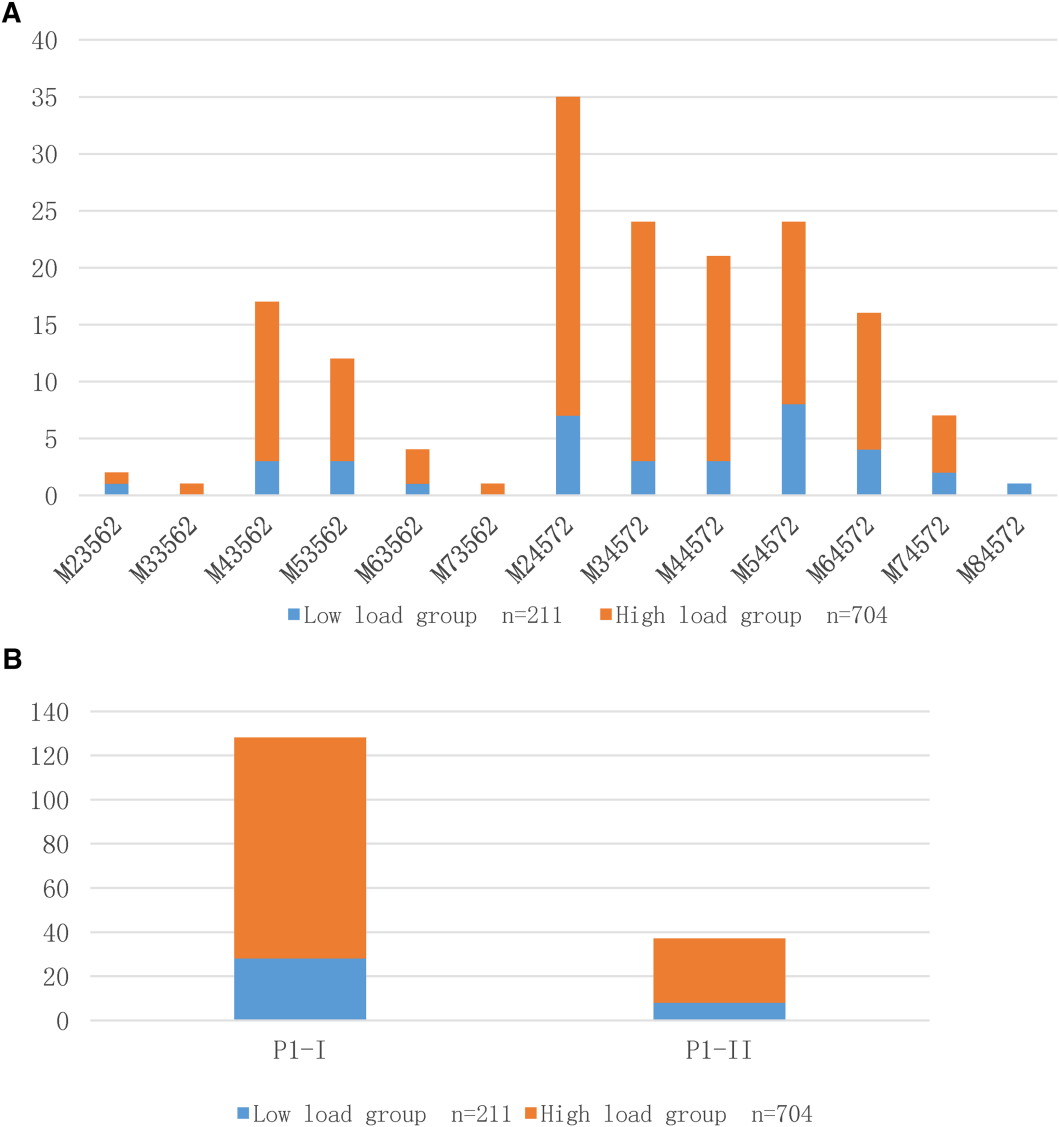
(**A**) Distribution of MP DNA load using MLVA typing method; (**B**) Distribution of MP DNA load using RFLP typing method.

After 165 MP DNA-positive BALF specimens were isolated and cultured to confirm their survival, they were genotyped using P1-RFLP and multiple-locus variable number tandem repeat analysis (MLVA). The PI-I type dominated the different load groups. Among the MLVA subtypes, except for M84572, which was dominated by children in the low-load group, M23562 in the low and high-load groups, respectively, accounted for one exception, and the high-load group dominated the other types. However, there was no statistically significant difference between the groups with different DNA loadings and MP genotyping (Table 1). Figure 1 shows the distribution of MP DNA loads between the two genotyping methods. In the low-load group, M54572 (22.2%), M24572 (19.4%), and M64572 (11.1%) were dominant, and M33562 and M73562 were not detected, whereas M24572 (21.7%), M34572 (16.3%), and M44572 (14.0%) were dominant and M84572 was not detected.

**Table 1:** MP genotyping with different DNA loadings.

Typing	Total number		DNA load [*n* (%)]		*χ* ^2^	*P*
			Low-load group *n* = 36	High-load group *n* = 129		
MLVA						
M3-5-6-2	**37**		**8 (22.2)**	**29** (**22.5)**	9.245	0.682
M23562	B	2	1 (2.8)	1 (0.8)		
M33562	G	1	0	1 (0.8)		
M43562	M	17	3 (8.3)	14 (10.9)		
M53562	S	12	3 (8.3)	9 (7.0)		
M63562	V	4	1 (2.8)	3 (2.3)		
M73562	Y	1	0	1 (0.8)		
M4-5-7-2	**128**		**28 (77.8)**	**100** (**77.5)**		
M24572	E	35	7 (19.4)	28 (21.7)		
M34572	J	24	3 (8.3)	21 (16.3)		
M44572	P	21	3 (8.3)	18 (14.0)		
M54572	U	24	8 (22.2)	16 (12.4)		
M64572	X	16	4 (11.1)	12 (9.3)		
M74572	Z	7	2 (5.5)	5 (3.9)		
M84572	a	1	1 (2.8)	0		
P1						
P1-I	128		28 (77.8)	100 (77.5)	0.001	0.974
P1-II	37		8 (22.2)	29 (22.5)		

Bold values represent the total number of each group.

### 3.3 Comparison of general information and clinical features

Children with MP infection had a cough, and 98.1% and 99.6% of children in the low- and high-load groups had a fever. There were 45 (21.3%) and 211 (30.0%) children with pneumonia diagnosed as severe and refractory MPP in the low and high-load groups, respectively, and the difference was statistically significant (*χ*^2 ^= 6.021, *P *< 0.05).

The children in the low-load group had a higher duration of fever and cough than those in the high-load group, and more children had wheezing and shortness of breath; however, the children in the high-load group had a higher length of hospitalization and maximum fever, and higher incidence of chills/chilliness and abdominal pain than those in the low-load group. The difference was statistically significant, as shown in Table 2.

**Table 2:** Comparison of general information and clinical characteristics of children after MP infection with different DNA loads.

Basic information	DNA load			
	Low-load group	High-load group	*χ* ^2^ */Z*	*P*
	*n* = 211	*n* = 704		
Male [*n* (%)]	109 (51.7)	356 (50.6)	*χ*^2 ^= 0.077	*P = *0.781
Age [Years of age, M (Q_1_, Q_3_)]	6.0 (4.0, 8.0)	6.0 (4.0, 8.0)	*Z *= −0.286	*P = *0.388
Clinical features				
Use of macrolides before admission [*n* (%)]	148 (70.1)	458 (65.1)	*χ*^2 ^= 1.877	*P = *0.171
Duration of macrolide use before admission [d, M (Q_1_, Q_3_)]	3.0 (2.0, 4.0)	3.0 (2.0, 4.0)	*Z *= −1.680	*P *= 0.093
Use of glucocorticoids before admission [n (%)]	56 (26.5)	146 (20.7)	*χ*^2 ^= 3.176	*P = *0.075
Length of hospitalization [d, M (Q_1_, Q_3_)]	6.0 (5.0, 8.0)	7.0 (6.0, 9.0)	***Z *= −6.337**	***P *= 0.000**
Maximum fever temperature [°C, M (Q_1_, Q_3_)]	39.5 (39.0, 39.9)	39.5 (39.0, 40.0)	***Z *= −1.800**	***P *= 0.036**
37.5–38°C	6 (2.8)	7 (0.1)	*χ*^2 ^= 4.252	*P *= 0.236
38.1–39°C	50 (23.7)	170 (24.1)		
39.1–41°C	142 (67.3)	512 (72.7)		
>41°C	1 (0.5)	7 (0.1)		
Fever duration [d, M (Q1, Q3)]	6.0 (4.0, 9.0)	6.0 (4.0, 7.0)	***Z *= -2.522**	***P *= 0.006**
Cough Duration [d, M (Q1, Q3)]	7.0 (4.0, 10.0)	5.0 (4.0, 7.0)	***Z *= −4.782**	***P *= 0.000**
Cough symptoms [*n* (%)]				
Excessive phlegm	163 (77.3)	578 (82.1)	*χ*^2 ^= 3.994	*P *= 0.136
Less phlegm	32 (15.2)	95 (13.5)	* *	* *
Dry cough	16 (7.6)	31 (4.4)	* *	* *
Other symptoms [*n* (%)]				
Wheezing	12 (5.7)	11 (1.6)	***χ*^2 ^= 11.271**	***P *= 0.001**
Shortness of breath	11 (5.2)	14 (2.0)	***χ*^2 ^= 6.352**	***P *= 0.012**
Chills/chilliness	25 (11.8)	131 (18.6)	***χ*^2 ^= 5.245**	***P *= 0.022**
Twitch	0	5 (0.7)	*χ*^2 ^= 0.483	*P *= 0.487
Vomiting	44 (20.9)	164 (23.3)	*χ*^2 ^= 0.551	*P *= 0.458
Abdominal pain	14 (6.6)	100 (14.2)	***χ*^2 ^= 8.528**	***P *= 0.003**
Diarrhea	12 (5.7)	44 (6.3)	*χ*^2 ^= 0.089	*P *= 0.765
Rash	11 (5.2)	26 (3.7)	*χ*^2 ^= 0.967	*P *= 0.325
Runny nose	7 (3.3)	16 (2.3)	*χ*^2 ^= 0.723	*P *= 0.395
Dizziness/headache	11 (5.2)	30 (4.3)	*χ*^2 ^= 0.344	*P *= 0.558
Pharyngeal congestion	208 (98.6)	702 (99.7)	*χ*^2 ^= 2.056	*P *= 0.152
Pharyngolaryngeal enlargement degree I	98 (46.4)	327 (46.4)	*χ*^2 ^= 0.000	*P *= 0.999
Pharyngolaryngeal enlargement degree II	23 (10.9)	93 (13.2)	*χ*^2 ^= 0.782	*P *= 0.376

Bold values represent statistical differences.

### 3.4 Comparison of laboratory indicators

After analysis (Table 3), the differences in white blood cell count (WBC), proportion of monocytes (MONO), and C-reactive protein (CRP), procalcitonin (PCT), and aspartate aminotransferase (AST) levels were statistically significant after infection with different MP loads. Children in the low-load group had relatively higher WBC and MONO levles; whereas those in the high-load group had relatively higher CRP, PCT, and AST levels.

**Table 3:** Comparison of laboratory parameters in children after MP infection with different DNA loads.

Laboratory metrics	DNA load [M (Q1, Q3)]		*Z*	*P*
	Low-load group	High-load group		
WBC [×10^9 ^/L, M (Q1, Q3)]	8.5 (7.2, 11.2)	8.0 (6.5, 9.7)	**−3**.**648**	**0**.**000**
NEUT [%, M (Q1, Q3)]	63.7 (56.3, 70.6)	62.6 (55.7, 69.5)	−0.534	0.297
LYMPH [%, M (Q1, Q3)]	25.8 (20.2, 32.5)	27.3 (20.9, 33.8)	−0.784	0.217
MONO [%, M (Q1, Q3)]	8.0 (6.2, 10.1)	7.5 (5.9, 9.6)	**−2**.**216**	**0**.**014**
CRP [mg/L, M (Q1, Q3)]	11.7 (7.3, 26.5)	16.1 (8.0, 30.0)	**−2**.**018**	**0**.**022**
PCT [ng/ml, M (Q1, Q3)]	0.10 (0.05, 0.21)	0.13 (0.08, 0.24)	**−3**.**501**	**0**.**000**
Fer [ng/ml, M (Q1, Q3)]	138.9 (91.1, 212.8)	140.1 (94.2, 228.0)	−0.509	0.306
IL-6 [pg/ml, M (Q1, Q3)]	16.0 (7.2, 28.4)	24.5 (15.2, 43.7)	−0.663	0.254
LDH [U/L, M (Q1, Q3)]	367.5 (309.0, 490.5)	393.0 (314.0, 530.0)	−1.415	0.079
ALT [U/L, M (Q1, Q3)]	15.0 (11.0, 21.5)	14.0 (11.0, 22.0)	−0.081	0.468
AST [U/L, M(Q1, Q3)]	28.0 (21.0, 34.8)	31.0 (25.0, 42.0)	**−4**.**762**	**0**.**000**

Bold values represent statistical differences.

### 3.5 Comparison of imaging performance

Notably, all children underwent chest computed tomography (CT) before admission or during hospitalization. Table 4 shows that the incidence of pleural effusion was higher in the children in the high-load group than those in the low-load group.

**Table 4:** Comparison of imaging manifestations in children after MP infection with different DNA loads.

DNA load	Radiological manifestations [*n* (%)]		
	Pleurisy *n* = 223	Pulmonary atelectasis *n* = 90	Pleural effusion *n* = 171
Low-load group *n* = 211	55 (26.1)	20 (9.5)	26 (12.3)
High-load group *n* = 704	168 (23.9)	70 (9.9)	145 (20.6)
*χ* ^2^	0.427	0.039	**7.314**
*P*	0.513	0.842	**0.007**

Bold values represent statistical differences.

### 3.6 Comparison of clinical diagnoses

After reviewing the relevant medical records of all children, the main clinical diagnoses were classified as respiratory system infections, including (1) pneumonia; (2) bronchopneumonia; (3) bronchiectasis; (4) asthma; (5) lung abscess, emphysema, pneumothorax, chest abscess, and hemothorax; (6) pertussis-like syndrome; (7) rhinitis and sinusitis; (8) laryngitis, tonsillitis, pharyngitis, eustachian tube inflammation, stomatitis, otitis media; and (9) conjunctivitis. Extrapulmonary complications are classified as: (1) cardiac system complications (including abnormal myocardial enzyme profile, myocardial injury, pericardial effusion, and heart failure); (2) neurological complications [including intracranial infection, hypoxemia, hypochlorhydria, hypo (hyper) potassiumemia, hyponatremia, hypocalcemia, hyperlactatemia, toxic encephalopathy, and convulsions]; (3) hematologic system complications (including neutropenia (deficiency), eosinophilia, thrombocytosis (reduction), and anemia); (4) urinary complications (including urinary tract infection, hematuria, proteinuria, acute pyelonephritis, primary nephrotic syndrome, ascites, glomerulonephritis, and bilateral testicular syringomyelia); (5) digestive complications (including hepatic impairment, liver function abnormalities, gastroenteritis, diarrheal disease, enterocolitis, intestinal bruising, hyperbilirubinemia, and jaundice); (6) skin system complications (including erythema multiforme, rash, urticaria, and eczema); (7) other diagnoses (including systemic inflammatory response syndrome, and sepsis).

The results showed that the overall incidence of respiratory infections and extrapulmonary complications was higher in the high-load group than in the low-load group. The odds of developing bronchitis, neurological complications, and digestive complications were higher in children in the low-load group than those in the high-load group (Table 5).

**Table 5:** Comparison of clinical diagnoses in children after MP infection with different DNA loads.

Clinical diagnoses	DNA load		*χ* ^2^	*P*
	Low-load group (*n* = 211)	High-load group (*n* = 704)		
Respiratory system infections	28 (13.2)	137 (19.5)	**4** **.** **208**	**0**.**040**
Bronchiectasis	19 (9.0)	115 (16.3)	**6**.**979**	**0**.**008**
Extrapulmonary complications	17 (8.1)	135 (19.2)	**14**.**490**	**0**.**000**
Neurological complications	1 (0.5)	45 (6.4)	**11**.**909**	**0**.**001**
Digestive complications	4 (1.9)	37 (5.3)	**4**.**282**	**0**.**039**

Bold values represent statistical differences.

### 3.7 Analysis of the degree of correlation and association of MP DNA load with clinical features and laboratory indicators

Spearman's correlation coefficient was used to analyze the correlation and degree of association between MP DNA load and the above quantitative indicators in children with MPP (Tables 6, 7).

**Table 6:** Analysis of the correlation and degree of association between MP DNA load and clinical characteristics.

	Length of hospitalization	Maximum fever temperature	Fever duration	Cough duration
Correlation coefficient r	0.246	0.088	−0.14	−0.203
*P*	**0**	**0**.**008**	**0**	**0**

Bold values represent statistical differences.

**Table 7:** Correlation and degree of association analysis between MP DNA load and laboratory indicators.

	WBC	NEUT	LYMPH	MONO	CRP	PCT	Fer	IL-6	LDH	ALT	AST
Correlation coefficient r	−0.126	−0.003	0.048	−0.102	0.079	0.127	0.01	0.209	0.034	0.003	0.15
*P*	**0**	0.925	0.147	**0**.**003**	**0**.**022**	**0**	0.756	**0**	0.306	0.931	**0**

Bold values represent statistical differences.

Spearman correlation coefficient analysis suggested that the MP DNA load was positively correlated with length of hospitalization, maximum fever temperature, CRP, PCT, interleukin-6 (IL-6), and AST levels, and negatively correlated with fever duration, cough duration, WBC count, and the proportion of MONO. Therefore, the higher the MP DNA copy number, the longer the hospitalization duration, the higher the maximum fever temperature, the higher the values of CRP, PCT, IL-6, and AST, and the shorter the duration of fever and cough, and the lower the WBC count and the proportion of MONO. The present results suggest that the degree of correlation is in the following order, from strongest to weakest: length of hospitalization > IL-6 > cough duration > AST > fever duration > PCT > WBC > proportion of MONO > maximum fever temperature > CRP levels.

Tables 2, 3 show that the length of hospitalization, maximum fever temperature, CRP, PCT, and AST levels were positively correlated with MP DNA load in Spearman correlation coefficients and statistically significant in the analysis of variance (ANOVA) between the load groups, suggesting a strong correlation. Therefore, a high MP DNA load suggests that the child will have a higher fever peak, longer hospital stay, and higher CRP, PCT, and AST levels. IL-6 showed a linear correlation with MP DNA load in Spearman's correlation coefficient. However, the difference between the MP DNA load groups was not statistically significant, suggesting a weak correlation, and further studies and analyses are needed.

## 4 Discussion

MP is one of the most important pathogens causing human respiratory infections, with widespread population susceptibility, periodic cluster epidemics occurring every 3–7 years, and infection rates as high as 50%–80% during peak epidemiologic periods or in limited spaces, with preschool and school-age children having the highest rates of infection. The disease has a slow onset and can be latent in the respiratory tract of infected patients, causing a prolonged disease course and the clinical symptoms after infection are diverse, with heavy symptoms and light signs leading to a high tendency to underestimate the disease upon imaging; therefore, it is particularly essential to correctly understand its clinical features and treatment methods.

Notably, several studies have confirmed that severe MP infections and refractory cases are significantly associated with MP pathogenicity and individual host immune response (3, 5). Therefore, studying the relationship between MP pathogenicity and the degree of host inflammatory and immune response status is essential to improve the treatment rate and quality of survival in patients with severe and/or refractory MP infections. Waites et al. (24) suggested that the local MP load responds to the balance between pathogen and host immune clearance; therefore, effective access to the biological properties of *in situ* MP infection is critical to achieving this theoretical study. This study aimed to analyze the association between MP DNA load in the BALF and MP typing, laboratory indicators, imaging manifestations, and clinical phenotypes to provide a basis for clinical disease assessment and MPP diagnostic protocols.

Previous studies have found that Mpn13, Mpn14, Mpn15, and Mpn16 strains exhibiting 4-5-7-2 are mostly P1-I type strains, whereas strains exhibiting 3-5-6-2 are mostly P1-II type strains. In this study, 128 and 37 cases, respectively, supported this hypothesis (25). However, the relationship between MP genotype and DNA load has rarely been reported. Nilsson et al. (10) found a correlation between the clinical severity of MP infection and DNA load but found no significant correlation with its subtype. However, their study performed only one type of typing, PCR-RFLP, for MP P1 adhesion protein, and most of the study population was selected from young and middle-aged people. The relationship between the MP genotype and DNA load in children needs further exploration, considering that children are not a reduced version of adults and have different development and morbidity types. In the 165 cases of BALF successfully cultured in this study, the typing results showed no statistical difference in the distribution of P1 and MLVA genotypes among the different MP DNA load samples, which is consistent with the report of Nilsson et al., who confirmed no significant association between MP subtypes and DNA load from the perspective of MP infection in adults.

MP invading the respiratory tract can directly activate the host inflammatory response, causing increased secretion of multiple inflammatory mediators, resulting in organismal damage and participation in developing severe and refractory MPP (3, 5). This study found that the CRP, PCT, and AST levels in children in the high-load group were significantly higher than those of children in the low-load group. A linear trend correlation analysis using Spearman's correlation coefficients also showed a positive correlation between MP DNA load and these serum biochemical indices, suggesting that MP DNA load is correlated with inflammatory response. Previous studies have confirmed that CRP, a non-specific systemic inflammatory response protein, reflects the severity of acute temporal pneumonia and extrapulmonary tissue damage (24, 26). PCT is a calcitonin precursor secreted by the thyroid gland. It is present in small amounts in an organism's normal state, and its level increases linearly when infected (27). Notably, some studies have suggested that both inflammatory mediators are closely associated with MPP and are critical biomarkers for measuring MP infection status and predicting the occurrence of severe and refractory MPP (28, 29). Serum CRP and PCT levels are lower after MP infection than after bacterial infection; however, they help predict severe MPP (28). The present study found that CRP and PCT levels were higher in children in the high-load group than in those in the low-load group, and the difference was statistically significant. MP DNA load was positively correlated with inflammatory response. The positive correlation between MP DNA load and inflammatory response suggests that in clinical practice, anti-inflammatory therapy should be strengthened early in patients with a high load during infection to avoid the excessive release of inflammatory mediators or autoimmune imbalance leading to disease exacerbation and drug resistance. Among the other biochemical indicators, increased AST was significant, associated with an increased rate of digestive system comorbidity; however, alanine aminotransferase (ALT), which reflects the characteristics of liver function, did not suggest specific changes. This was analyzed because AST can be detected in various body tissues and is not tissue-specific; for example, mildly hemolyzed erythrocytes, myocardial infarction, and cardiac arrhythmia can cause abnormal increases in AST. Therefore, this study aimed to improve other relevant experimental data to clarify their application in diagnosing MP infections.

This study showed that children in the low-load group had significantly higher cough duration, wheezing frequency, and shortness of breath than those in the high-load group, and the WBC and MONO counts were significantly increased in the low-load group. This may be because the increase in WBC and MONO counts in the low-load group and lesions are mainly concentrated in the airway, which may promote the accumulation of airway epithelial cells and the release of several inflammatory factors, which trigger airway hyperresponsiveness and lead to shortness of breath and wheezing in children.

Children in the high-load group had longer hospital stays and higher maximum fever temperatures, which resulted in a higher incidence of chills. The Spearman correlation coefficients showed the same trend, suggesting that this may be associated with the difficulty in clearing overloaded MP in the body and the persistent immune imbalance in children. In clinical practice, it is necessary to extend the period of relevant drug therapy and strengthen immune-related treatments. Local high MP load infection can directly damage bronchial epithelial cells, resulting in epithelial cell necrosis, reduced or even absent cilia, and impaired secretion drainage, which aggravates poor local toxin clearance, forming a vicious circle, leading to bronchial mucosal erosion, secretion obstruction, plastic sputum formation, and local ischemia and hypoxia, resulting in epithelial cell repair dysfunction, end small vessel fibrosis, ischemic and hypoxic injury of small airways, irreversible fibrotic lesions and airway obstruction. This leads to pulmonary atelectasis, bronchial occlusion, increased local inflammatory response, severe damage to lung tissue, and peripheral respiratory failure, which can significantly increase invasive operations such as tracheal intubation and mechanical ventilation and seriously affect prognosis (17, 30).

In addition to pulmonary inflammation, MP infection can cause multi-organ damage through immune cascade reactions mediated by multi-pathway mechanisms, such as multisystem lesions in the skin and digestive, urinary, hematological, neurological, and circulatory systems (31, 32), and in severe cases, acute pericardial tamponade, hemolytic uremic syndrome, and ischemic stroke (33, 34). This is due to the presence of partially common antigens between MP antigens and specific body tissues (heart, liver, lung, brain, kidney, and smooth muscle), which can induce the body to produce autoantibodies such as Immunoglobulins G and E, inducing autoimmune reactions and causing damage to body tissues with the same antigenic structure, which can lead to damage to other target organs outside the respiratory tract and extrapulmonary complications in multiple systems (35–37).

In this study, children in the high-load group were more likely to have imaging changes, such as pleural effusion, and the incidence of respiratory infections and extrapulmonary complications was higher than that of children in the low-load group. MP infection causes a double blow to extrapulmonary organs by direct damage and an indirect immune response, causing multi-organ damage to the primary lung and extrapulmonary tissues. Therefore, we hypothesized that a high MP load could predict the severity of airway and extrapulmonary damage in children and provide data to support early disease intervention.

In conclusion, MP DNA load was not significantly correlated with MP typing but was significantly correlated with clinical manifestations, laboratory parameters, and imaging characteristics in children. Therefore, monitoring MP load can provide reasonable, economical, and efficient reference suggestions for the diagnosis and severity assessment of children with MP infection, which can help monitor and predict the disease and guide the rational use of clinical drugs to effectively improve children's prognosis, survival rate, and quality of life.

## Data Availability

The raw data supporting the conclusions of this article will be made available by the authors, without undue reservation.
